# A predictive model for intraabdominal infection after radical gastrectomy in elderly patients

**DOI:** 10.1097/MD.0000000000037489

**Published:** 2024-03-15

**Authors:** Xiaohan Yu, Wanyun Tang, Chenglin Bai, Runzhuo Li, Bo Feng, Jinge Wu, Xianzhan Guo, Hong Chen, Meng Li

**Affiliations:** a General Surgery Department, Dandong Central Hospital, China Medical University, Dandong, Liaoning, China; b Orthopedics Department, Dandong Central Hospital, China Medical University, Dandong, Liaoning, China; c General Surgery Department, Dandong Central Hospital, Jinzhou Medical University, Dandong, Liaoning, China; d Gastroenterology Department, Dandong Central Hospital, China Medical University, Dandong, Liaoning, China.

**Keywords:** elderly, gastric cancer, intraabdominal infection, nomogram, radical gastrectomy

## Abstract

Gastric cancer (GC) is one of the most common malignant tumors worldwide and the fourth leading cause of cancer-related deaths, with a relatively high incidence among the elderly population. Surgical resection is the mainstay treatment for GC and is currently the only cure. However, the incidence of postoperative intraabdominal infections remains high and seriously affects the prognosis. This study aimed to explore the risk factors for intraabdominal infections after radical gastrectomy in elderly patients and to establish and validate a risk prediction model. We collected the clinical data of 322 GC patients, who underwent radical gastrectomy at the General Surgery Department of China Medical University Dandong Central Hospital from January 2016 to January 2023. The patients were divided into an infected group (n = 27) and a noninfected group (n = 295) according to whether intraabdominal infections occurred postoperatively. A nomogram risk prediction model for the occurrence of postoperative intraabdominal infections was developed. All patients were randomized into a training set (n = 225) and a validation set (n = 97) in a 7:3 ratio, and the model was internally validated. Of the 322 patients, 27 (8.3%) experienced postoperative intraabdominal infections. Single-factor analysis revealed associations of intraabdominal infection with body mass index, glucose, hemoglobin, albumin, and other factors. The multifactorial analysis confirmed that body mass index, glucose, hemoglobin, albumin, surgical duration, and bleeding volume were independent risk factors for intraabdominal infections. The nomogram constructed based on these factors demonstrated excellent performance in both the training and validation sets. A nomogram model was developed and validated to predict the risk of intraabdominal infection after radical gastrectomy. The model has a good predictive performance, which could help clinicians prevent the occurrence of intraabdominal infections after radical gastrectomy in elderly patients.

## 1. Introduction

Gastric cancer (GC) is one of the most common malignant tumors worldwide and the fourth leading cause of cancer-related deaths.^[1]^ Surgical resection is the mainstay of treatment for GC and is currently the only cure.^[2]^ The incidence of GC is relatively high in the elderly population, and its risk gradually increases with age, especially among patients aged >60 years. Elderly patients usually have poor physical condition, poor nutritional intake, and comorbidities, such as heart disease, diabetes mellitus, and hypertension, which may increase the risk of postoperative complications.^[3–5]^ Although mortality and recurrence rates after radical gastrectomy have decreased with modern surgical advances, the incidence of postoperative intraabdominal infections remains relatively high.^[6,7]^ While Mao et al^[8]^ reported a postoperative intraabdominal infection rate of 8.9% in patients undergoing radical GC surgery, Zhang et al^[9]^ reported a rate of 7.2%. Postoperative intraabdominal infection following radical gastrectomy significantly prolongs hospitalization and healthcare costs^[10]^ and impairs short-term postoperative survival.^[11,12]^ Moreover, the prolonged inflammatory response it induces has a negative impact on recurrence-free survival and overall survival.^[13]^ Postoperative infection can also promote tumor recurrence and metastasis.^[14,15]^ Research indicates that patients with complications of postoperative intraabdominal infections following radical GC surgery have a lower survival rate than those without intraabdominal infections.^[16]^ Currently, no specific study has been conducted to construct a nomogram predicting postoperative intraabdominal infections in elderly patients. Therefore, it is necessary to clarify the risk factors for infection after radical gastrectomy and take active preventive measures for high-risk elderly patients.

Univariate and multivariate logistic regression analyses were used to screen risk factors by collecting clinical data from elderly patients who underwent radical gastrectomy at our center. We developed and validated a nomogram model to predict the risk of postoperative intraabdominal infections in elderly patients undergoing radical gastrectomy. The model exhibited excellent predictive performance and could serve as a valuable guide for the clinical prevention of postoperative intraabdominal infections in elderly patients.

## 2. Methods

### 2.1. Study design

We retrospectively collected the data of patients who underwent radical gastrectomy at the China Medical University Dandong Central Hospital from January 2016 to January 2023. The inclusion criteria were as follows: age ≥ 60 years, postoperative pathologic diagnosis of gastric adenocarcinoma, radical resection performed, and complete clinicopathological data available. The exclusion criteria were as follows: severe cardiac, hepatic, or pulmonary insufficiency; emergency surgery owing to acute obstruction, perforation, or other reasons; palliative resection for extensive metastases; combined resection of multiple organs; preoperative intraabdominal infection; and incomplete clinical data. Finally, 322 GC patients who met the inclusion criteria were enrolled in this study. Based on whether postoperative infection occurred, they were divided into an infected group (n = 27) and a noninfected group (n = 295).

### 2.2. Diagnostic criteria for infection

Postoperative intraabdominal infection was diagnosed strictly according to the following criteria of the Chinese Medical Association Guidelines^[17]^ for the Diagnosis and Treatment of Gastric Cancer: abdominal pain and signs of peritonitis associated with anastomosis as well as changes in laboratory indices; cloudy drainage fluid around the anastomosis, change in odor, or other suggestive changes; confirmation of peri-anastomotic abscess during secondary surgery; and abdominopelvic infection far from the anastomosis and considered unrelated to the anastomosis diagnosed on imaging or during secondary surgery.

### 2.3. Collection indicators

We collected data for the following parameters:

1. General information: age, sex, smoking history, drinking history, hypertension, pulmonary insufficiency, history of abdominal surgery, and body mass index (BMI).
2. Preoperative hematological indicators: hemoglobin (Hb), platelet count, white blood cell count, neutrophil (NE) count, lymphocyte (LY) count, albumin (Alb), serum creatinine, blood urea nitrogen, alanine aminotransferase, aspartate aminotransferase, lactate dehydrogenase, alkaline phosphatase, glucose (Glu), total bilirubin, triglycerides, total cholesterol, prothrombin time, activated partial thromboplastin time, thrombin time, systemic immune-inflammation index (platelet count × NE-to-LY ratio), prognostic nutritional index (serum Alb [g/L] + 5 × total peripheral blood LY [×109/L]), NE-to-LY ratio, and the controlling nutritional status score.
3. Surgery-related indicators: the extent of surgical resection, surgical duration, intraoperative fluid replacement volume, bleeding volume, tumor size, the American Society of Anesthesiologists (ASA) classification, clinical staging, and tumor differentiation level.
4. Other indicators: gastric tube retention time, urinary catheter retention time, number of drains, and duration of drain retention.

### 2.4. Surgical approach and perioperative management

All patients were operated on by our experienced surgeons according to the Japanese guidelines for GC treatment.^[18]^ Patients with cT1a or cT1b tumors without lymph node involvement or distant metastases underwent gastrectomy combined with D1 or D1 + lymph node dissection. Gastrectomy combined with D2 or D2 + lymph node dissection was performed for progressive GC (T2 or higher stages or positive lymph nodes). Routine fasting for 8 hours and bowel cleansing were performed a few hours before surgery. All patients were administered second-generation cephalosporins and metronidazole as prophylactic antibiotics 0.5 to 1 hour before surgery, and additional antibiotics were administered once when the operation lasted ≥3 h or the bleeding volume was ≥1500 mL. Prophylactic antibiotics were administered for no more than 24 hours. Routine administration of cephalosporins during the perioperative period for infection control was continued for 3 to 5 days postoperatively. If no drainage fluid was found, the drainage fluids were relatively clear within 2 to 3 days, and the patient had no abdominal pain, fever, or other uncomfortable symptoms, then the drainage tube was removed.

### 2.5. Statistical methods

Statistical analyses were performed using Statistical Product and Service Solutions (version 26.0). A normality test was performed initially for continuous data. All normally distributed data are presented as means ± standard deviations, followed by the application of an independent sample *t* test. Nonnormally distributed data are presented as medians and interquartile ranges. The data were compared using the Mann–Whitney *U* test. Categorical data were analyzed using the χ² test or Fisher exact test. Variables with *P* values <.05 in the univariate analysis were included in the binary logistic regression for multivariate analysis, aiming to identify independent risk factors for intraabdominal infection. R software (version 4.0.5) was used to construct an individualized nomogram prediction model to predict the occurrence of intraabdominal infections. Discrimination was assessed using receiver operating characteristics (ROC) curves and the C-index. The calibration accuracy of the model was evaluated by plotting calibration curves, and a decision curve analysis (DCA) was conducted to assess the clinical utility of the model across various threshold probabilities.

## 3. Results

### 3.1. Intraabdominal infections occurring after surgery for gastric cancer

**Figure 1.**
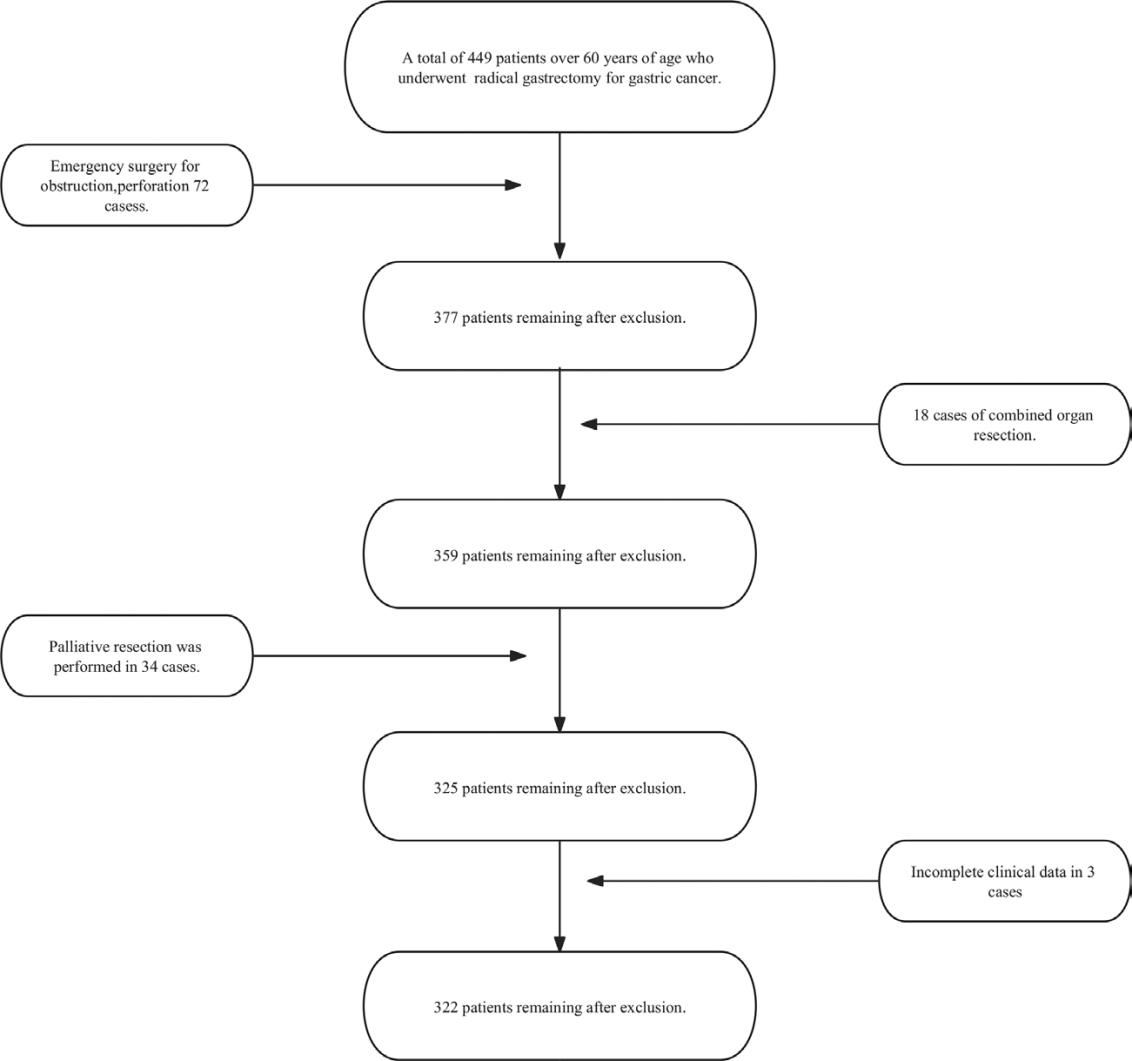
Flowchart of 322 elderly patients with gastric cancer undergoing radical gastrectomy.

This study included 322 patients who underwent radical gastrectomy, comprising 220 males and 102 females, with a minimum age of 60 years, a maximum age of 88 years, and an average age of 66.5 years (Fig. 1). Among them, 27 (8.3%) patients developed intraabdominal infections during the postoperative period.

### 3.2. Single-factor analysis of postoperative intraabdominal infections

Univariate analysis showed that the infected and noninfected groups differed statistically in the BMI, Glu, Hb, blood urea nitrogen, Alb, prothrombin time, prognostic nutritional index, surgical duration, bleeding volume, American Society of Anesthesiologists classification, gastric tube retention time, urinary catheter retention time, and drain retention time (*P* < .05) (Table 1).

**Table 1:** Univariate analysis of factors affecting abdominal infection after radical gastrectomy.

Variable	Total patients (n = 322)	The noninfected group (n = 295)	The infected group (n = 27)	Statistical value	*P*
**General information**					
Age (years), median (Q1, Q3)	66.5 (63, 71)	66 (63, 71)	68 (63, 71)	–0.341	.733
Gender, n (%)				1.217	.27
Female	102 (32)	96 (33)	6 (22)		
Male	220 (68)	199 (67)	21 (78)		
Smoking history, n (%)				0.144	.704
No	248 (77)	228 (77)	20 (74)		
Yes	74 (23)	67 (23)	7 (26)		
Drinking history n (%)				0.916	.338
No	266 (83)	246 (83)	20 (74)		
Yes	56 (17)	49 (17)	7 (26)		
Hypertension, n (%)				0.387	.534
No	234 (73)	213 (72)	21 (78)		
Yes	88 (27)	82 (28)	6 (22)		
Pulmonary insufficiency, n (%)				0	.993
No	304 (94)	278 (94)	26 (96)		
Yes	18 (6)	17 (6)	1 (4)		
History of previous abdominal surgery, n (%)				0.225	.635
No	283 (88)	258 (87)	25 (93)		
Yes	39 (12)	37 (13)	2 (7)		
BMI, median (Q1, Q3)	23 (22, 26)	23 (22, 26)	26 (23, 28)	–3.148	.002
**Preoperative hematological indicators**					
Hb, median (Q1, Q3)	124 (105, 134.75)	125 (106, 135)	101 (89, 123.5)	–3.795	<.001
PLT (×10^9^/L), median (Q1, Q3)	232 (193.25, 285.5)	229 (192, 283.5)	236 (199.5, 297)	–0.631	.528
WBC (×10^9^/L), median (Q1, Q3)	5.8 (4.9, 7.1)	5.9 (4.9, 7.1)	5.2 (5, 7.35)	–0.428	.669
NE (×109/L), median (Q1, Q3)	3.51 (2.7, 4.57)	3.52 (2.7, 4.5)	3.47 (2.8, 4.7)	–0.305	.761
LY (×109/L), median (Q1, Q3)	1.6 (1.2, 2)	1.6 (1.23, 2)	1.35 (1.08, 1.71)	–1.491	.136
Alb, median (Q1, Q3)	39 (35, 42)	40 (36, 43)	36 (27, 36.5)	–4.32	<.001
Scr (µmol/L), median (Q1, Q3)	66 (57.25, 76)	66 (58, 77)	67 (57, 72.5)	–0.541	.588
BUN (mmol/L), median (Q1, Q3)	5.6 (4.6, 7)	5.7 (4.65, 7.15)	4.9 (3.75, 5.7)	–2.926	.003
ALT (U/L), median (Q1, Q3)	15 (11, 21)	15 (11, 21)	14 (10, 22.5)	–0.138	.89
AST (U/L), median (Q1, Q3)	17 (14, 21)	17 (14, 21)	16 (14.5, 22)	–0.017	.986
LDH (U/L), median (Q1, Q3)	165 (144, 189)	165 (145, 188)	166 (142.5, 198)	–0.124	.901
ALP (U/L), median (Q1, Q3)	78 (65, 97)	78 (65, 97)	76 (69, 95)	–0.008	.994
GLU (mmol/L), median (Q1, Q3)	5.4 (4.9, 6.2)	5.4 (4.85, 6.1)	5.8 (5.4, 8.3)	–2.808	.005
Tbil (µmol/L), median (Q1, Q3)	11 (8, 14.9)	11 (8, 15)	12 (8, 14.2)	–0.137	.891
TG (mmol/L), median (Q1, Q3)	1.24 (0.93, 1.79)	1.25 (0.93, 1.79)	1.18 (1.01, 1.55)	–0.446	.656
TC (mg/dL), median (Q1, Q3)	166.92 (142.31, 194.23)	166.92 (142.69, 197.69)	166.15 (128.27, 181.15)	–1.219	.223
PT(s), median (Q1, Q3)	12.1 (11.4, 13)	12.1 (11.4, 13)	12.6 (12.15, 13.25)	–1.977	.048
APTT(s), median (Q1, Q3)	29.7 (27.42, 32.1)	29.7 (27.45, 32.05)	29.7 (27.55, 32.6)	–0.078	.938
TT(s), median (Q1, Q3)	14.8 (13.7, 15.9)	14.8 (13.7, 15.9)	15 (14.1, 16.1)	–0.685	.493
SII, median (Q1, Q3)	508.85 (344.27, 874.89)	508.35 (344.45, 808.85)	553.02 (347.82, 1039.44)	–0.85	.395
PNI, median (Q1, Q3)	48.33 (43.5, 52)	48.5 (43.5, 52.45)	44.05 (39.25, 49.47)	–2.429	.015
NLR, median (Q1, Q3)	2.21 (1.59, 3.26)	2.17 (1.58, 3.18)	2.41 (1.6, 3.77)	–0.915	.36
COUNT score, n (%)				2.98	.084
<5 points	256 (80)	238 (81)	18 (67)		
≥5 points	66 (20)	57 (19)	9 (33)		
**Surgery-related indicators**					
Surgical resection extent, n (%)				0.001	.976
Major gastrectomy	269 (84)	247 (84)	22 (81)		
Total gastrectomy	53 (16)	48 (16)	5 (19)		
Surgical duration, median (Q1, Q3)	195 (160, 232.25)	190 (160, 230)	225 (187.5, 265)	–2.82	.005
Intraoperative fluid replacement volume, Median (Q1, Q3)	2000 (1500, 2500)	2000 (1500, 2500)	2000 (1500, 2375)	–0.468	.64
Bleeding volume, median (Q1, Q3)	200 (100, 400)	200 (100, 400)	400 (200, 550)	–3.144	.002
Tumour size (Q1, Q3)	4 (2.5, 6)	4 (2.5, 6)	5 (3, 6.5)	–1.056	.291
ASA classification, n (%)				4.483	.034
<3	254 (79)	237 (80)	17 (63)		
≥3	68 (21)	58 (20)	10 (37)		
Clinical staging, n (%)				2.896	.408
1	95 (30)	90 (31)	5 (19)		
2	72 (22)	65 (22)	7 (26)		
3	129 (40)	118 (40)	11 (41)		
4	26 (8)	22 (7)	4 (15)		
Tumour differentiation level, n (%)				3.008	.222
Low differentiation	164 (51)	146 (49)	18 (67)		
Middle differentiation	101 (31)	95 (32)	6 (22)		
High differentiation	57 (18)	54 (18)	3 (11)		
**Other indicators**					
Gastric tube retention time (days), median (Q1, Q3)	7 (6, 8)	7 (6, 8)	7 (6.5, 9.5)	–2.39	.017
Urinary catheter retention time (days), median (Q1, Q3)	2.5 (2, 4)	2 (2, 4)	3 (2, 4)	–2.287	.022
Number of drains (pcs), n (%)				0.015	.902
<3	271 (84)	249 (84)	22 (81)		
≥3	51 (16)	46 (16)	5 (19)		
Duration of drain retention (days), n (%)				4.943	.026
<14	265 (82)	247 (84)	18 (67)		
≥14	57 (18)	48 (16)	9 33)		

Alb = albumin , ASA = American Society of Anesthesiologists, BMI = body mass index, BUN = blood urea nitrogen, DCA = decision curve analysis, EPV = events per variable, Glu = glucose, Hb = hemoglobin, LY = lymphocyte, NE = neutrophil, NLR = neutrophil-to-lymphocyte ratio, PLT = platelet, PNI = prognostic nutritional index, PT = prothrombin time, ROC = receiving operating characteristics, WBC = white blood cell.

### 3.3. Multifactorial analysis of postoperative intraabdominal infections

Binary logistic regression analysis was performed with the occurrence of intraabdominal infection after radical gastrectomy as the dependent variable and variables with *P* values <.05 in the univariate analysis as the independent variables. The results of the analysis showed that the 6 variables, BMI, Glu, Hb, Alb, surgical duration, and bleeding volume, independently influenced the occurrence of intraabdominal infections after surgery (*P* < .05) (Table 2).

**Table 2:** Multivariate analysis of factors affecting abdominal infection after radical gastrectomy.

Variable	SE	Wald	OR	95% CI	*P*
BMI	0.098	6.016	1.271	1.049–1.540	.014
Glu	0.192	6.842	1.65	1.134–2.402	.009
Hb	0.014	10.628	0.956	0.931–0.982	.001
Alb	0.046	7.113	0.885	0.808–0.968	.008
Surgical duration	0.005	6.064	1.012	1.002–1.022	.014
Bleeding volume	0.001	5.88	1.003	1.001–1.005	.015

Alb = albumin, BMI = body mass index, Glu = glucose, Hb = hemoglobin.

### 3.4. Establishment and validation of the nomogram

**Figure 2.**
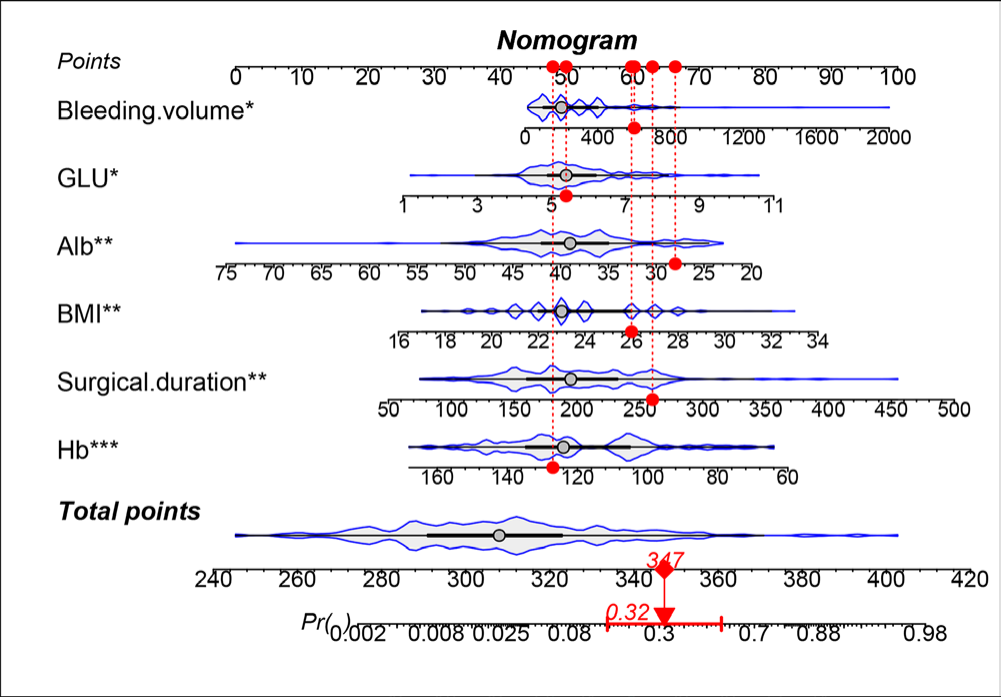
Nomogram for postoperative intraabdominal infections in elderly patients with gastric cancer. Alb = albumin, BMI = body mass index, Glu = glucose, Hb = hemoglobin.

**Figure 3.**
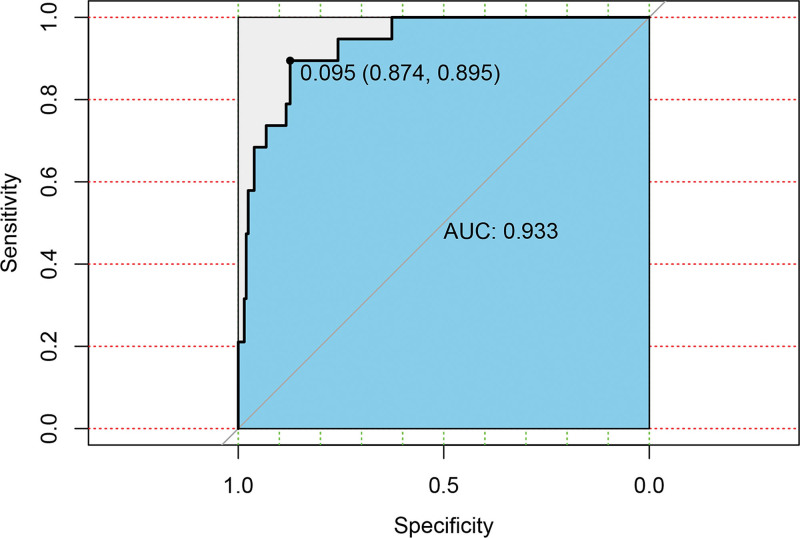
Receiver operating characteristics curve for the training group. AUC = area under the curve.

**Figure 4.**
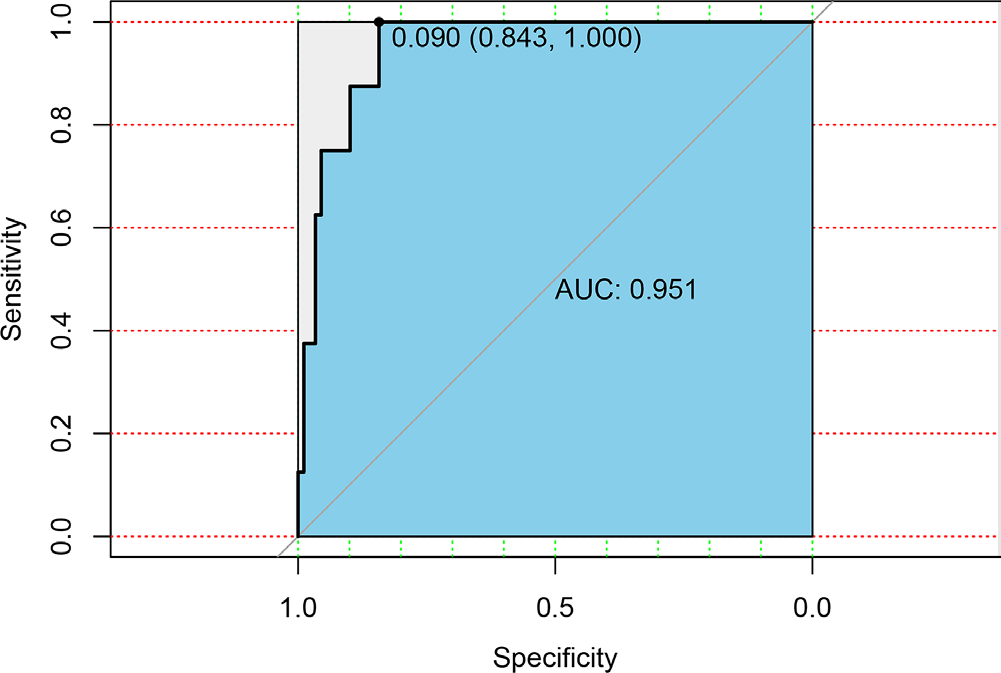
Receiver operating characteristics curve for the validation group. AUC = area under the curve.

**Figure 5.**
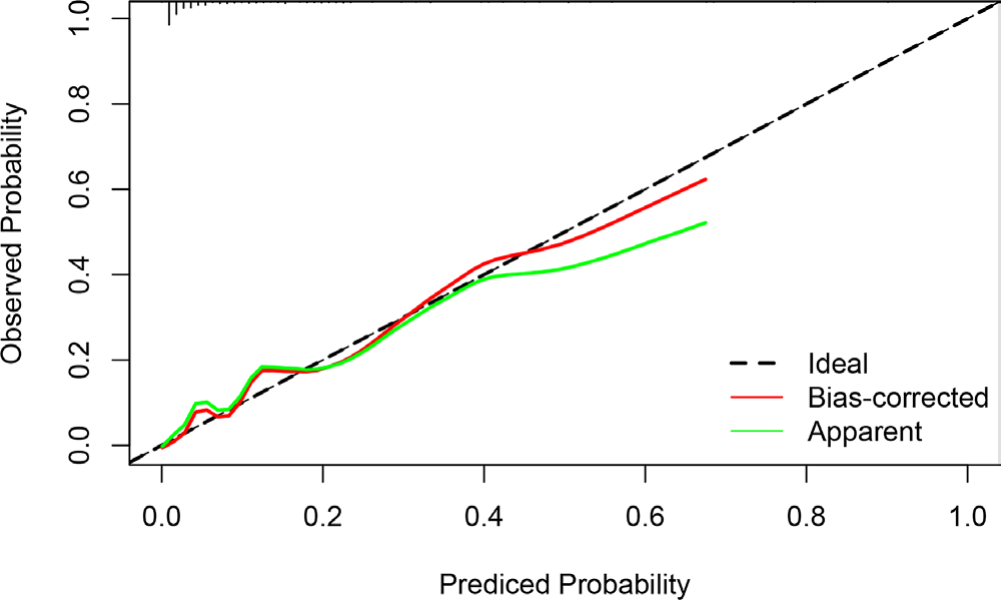
Decision curve analysis for the training group.

**Figure 6.**
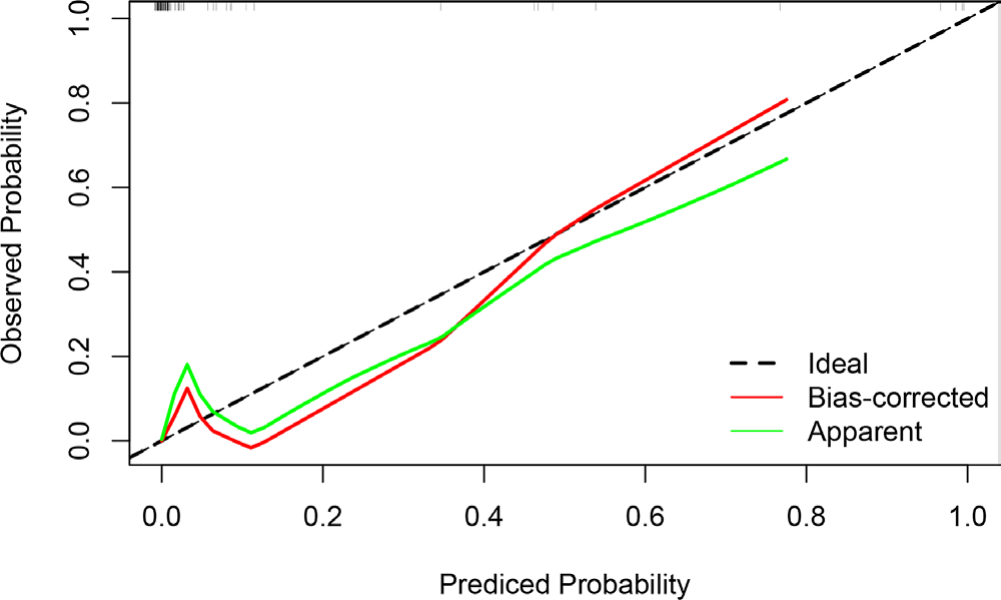
Decision curve analysis for the validation group.

**Figure 7.**
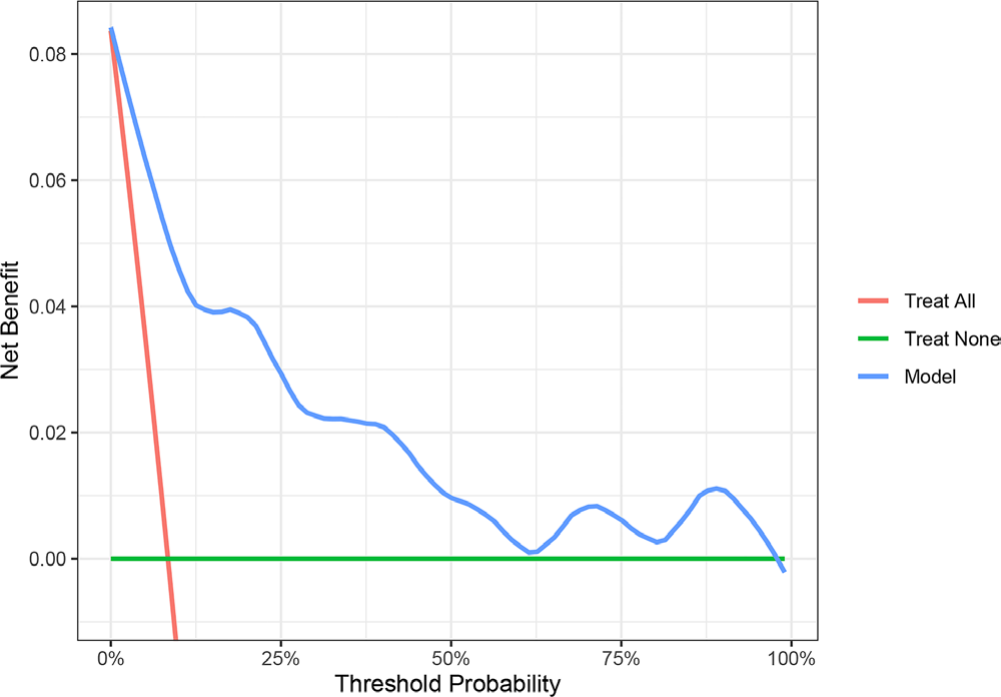
Decision curve analysis for predictive modeling.

Based on the independent influencing factors screened out in the multifactor analysis, a nomogram prediction model was constructed (Fig. 2). The ROC curve was plotted, and the area under the curve was 0.933 for the training cohort (n = 225) and 0.951 for the validation cohort (n = 97) (Figs. 3 and 4), indicating that the model showed good prediction ability in both the training and validation sets. Second, bootstrapping was used to sample the model 1000 times, and the calibration curves of the model were plotted (Figs. 5 and 6), which showed good agreement between the predicted and actual probabilities of occurrence of the model. Finally, DCA curves were plotted to assess the clinical application value of the model (Fig. 7). As shown in Figure 7, the horizontal axis is the high-risk threshold, and the vertical axis is the net benefit. As the threshold probability varies, the net benefit changes according to the model’s predicted value of the intervention, and the DCA curve suggests that the nomogram model has a good clinical utility value.

## 4. Discussion

As the fifth most common cancer worldwide, surgical resection is the primary treatment for stomach cancer.^[1,2]^ The risk of GC increases with age, and elderly patients are often more prone to postoperative complications than young patients because of their poor physical condition. Although postoperative complications after surgical resection in GC have decreased with advances in surgical techniques and perioperative management, intraabdominal infections remain one of the most serious postoperative complications of GC, often causing unnecessary discomfort to patients. Particularly, intraabdominal infections associated with the pancreas and anastomotic fistulas can lead to significantly prolonged hospital stay, septic shock, multi-organ failure, and even death.^[19]^ Currently, no studies have investigated the risk of intraabdominal infections after radical gastrectomy in elderly patients. We found that hyperglycemia, obesity, anemia, low protein levels, prolonged operation time, and high intraoperative bleeding are independent risk factors for intraabdominal infections after GC surgery in elderly patients. Therefore, we constructed a clinical prediction model for intraabdominal infection after radical gastrectomy, which was empirically verified to have a good prediction performance.

Hyperglycemia is an important risk factor for intraabdominal infections after radical gastrectomy. GC surgery usually involves extensive abdominal trauma, and hyperglycemia causes atherosclerosis, leading to a poor microcirculatory blood supply, which subsequently causes reduced healing capacity and slow anastomotic healing.^[20,21]^ Moreover, hyperglycemia may negatively affect the immune system, which causes defects in the innate immune response (including NE and macrophage dysfunction) and adaptive immune response (including T-cell dysfunction), resulting in reduced resistance to pathogen invasion and an increased risk of postoperative intraabdominal infection.^[13]^

High BMI was associated with a significantly higher risk of intraabdominal infection after radical gastrectomy compared to normal BMI, which corroborated the findings of previous studies.^[10,22–24]^ Excess visceral fat makes it difficult to locate the boundary between the pancreas and lymph nodes, which may lead to intraoperative pancreatic injury and increased incidence of pancreas-related infections and abdominal abscesses.^[22]^ In patients with excess visceral fat, the hypertrophied mesentery creates tension on the anastomosis, and anastomotic leakage may occur when the tension and pressure at the anastomotic site are excessive.^[25,26]^ Additionally, adipose tissue has fewer blood vessels, resulting in poor resistance to infection and reduced immunity and healing.^[23]^ Surgery in overweight patients is associated with prolonged operative time and increased blood loss owing to poor exposure at the surgical site. Increased blood loss and perioperative blood transfusions may also lead to increased infection rates.

Serum Alb is considered the most important parameter associated with the degree of malnutrition, and low protein levels can cause postoperative complications.^[27,28]^ Plasma proteins are ingested by mononuclear phagocytes in the body and are broken down by enzymes in the cells into amino acids, which are used to synthesize proteins in the tissues. The lack of essential amino acids in patients with low Alb levels affects the synthesis of collagen, which in turn affects the anastomosis healing.^[29,30]^ Hypoalbuminemia reduces the body’s ability to produce proteins, while hypoproteinemia impairs the immune system and increases the likelihood of anastomotic leakage.

In patients with anemia, the oxygen-carrying capacity of the blood is reduced, and protein synthesis is dependent on tissue oxygenation; therefore, anastomotic healing is disturbed when there is inadequate blood supply.^[31]^ Anemia may lead to an increased inflammatory response, and a prolonged state of immune inflammation may affect the normal functioning of the immune system and increase the risk of infection.^[32]^

Previous studies have reported an increased incidence of intraabdominal infections in patients with prolonged surgical times.^[33–36]^ Prolonged operative times indicate that the surgery is technically difficult, possibly because of high BMI, difficult lymph node clearance, or intraoperative medical injury, increasing the patient’s exposure time on the operating table, thus increasing the potential risk of postoperative infection. Second, the immune system, usually divided into the innate and adaptive immune systems, is critical to the patient’s postoperative recovery, and prolonged anesthesia may suppress the immune system, affecting wound healing and increasing the infection risk.^[37–39]^

Intraoperative bleeding is considered a risk factor for intraabdominal infection.^[40]^ Heavy bleeding can lead to disruption of the clean environment of the surgical, allowing more opportunities for bacteria to enter the wound. If the bleeding site is not cleaned up, the blood clot serves as a bacterial culture medium, increasing the risk of intraabdominal infection. Bleeding results in inadequate oxygen supply to the tissues, which may interfere with neovascularization and normal cellular metabolism, thereby delaying the healing of the surgical incision.^[41]^ Intraoperative blood loss results in decreased blood flow, regional hypoxia, and metabolic and microenvironmental changes that can stimulate an inflammatory response characterized by the release of proinflammatory cytokines (i.e., tumor necrosis factor-α and interleukin-6) and acute-phase proteins, which can lead to reduced cell-mediated immunity and systemic immunosuppression, increasing the patient’s risk of infection.^[42]^

A nomogram is a common tool used to assess prognosis and has the advantages of simplicity, precision, accuracy, and easy quantification of clinical indicators. By simply calculating the sum of the scores corresponding to each key variable to generate a numerical probability specific to a clinical event, it fulfills our need for biological and clinical predictive models and the pursuit of individualized medicine.^[43]^

Despite our predictive model serving as a good indicator for predicting intraabdominal infections following radical gastrectomy in elderly patients, our study has certain limitations. First, when developing a multivariate predictive model, the sample size is typically based on the ratio of the number of individuals with outcome events to the number of candidate predictor variables, known as events per variable (EPV). An EPV ≥ 10 is widely advocated as an empirical rule for multivariate logistic and Cox regression analyses. Due to the limited surgical volume at our center, our study did not meet the empirical guideline of EPV ≥ 10. However, our research was exploratory in nature; multiple regression was conducted after variable selection, and the odds ratio values and confidence intervals seemed reasonable. The goodness-of-fit results indicated that the model building was successful, although there was a lack of stability in this result. Nevertheless, the indicator characteristics show a certain degree of similarity with those of previous studies, suggesting reasonable reliability. Some studies have also mentioned that coverage and bias are not severe at 5 to 9 EPV, often comparable to 10 to 16 EPV.^[44]^ Second, this was a retrospective study; therefore, selection bias was inevitable, and no external validation was conducted. Hence, external validation of the model in time and space is warranted in the future to improve its clinical applicability.

## 5. Conclusion

BMI, Glu, Hb, Alb, surgical duration, and bleeding volume are important factors influencing intraabdominal infection after radical gastrectomy. The prediction model of the nomogram based on this model is simple and graphic and has good prediction efficacy after evaluating the calibration curves, ROC curves, and clinical decision curves. Therefore, it can effectively screen high-risk patients and guide clinicians to optimize the perioperative management of high-risk patients to reduce the incidence of postoperative intraabdominal infections.

## Acknowledgments

We would like to thank Editage (www.editage.com) for English language editing.

## Author contributions

**Conceptualization:** Xiaohan Yu, Meng Li.

**Data curation:** Wanyun Tang.

**Investigation:** Xianzhan Guo.

**Methodology:** Chenglin Bai, Runzhuo Li.

**Software:** Wanyun Tang, Bo Feng, Jinge Wu.

**Validation:** Hong Chen.

**Writing – original draft:** XiaoHan Yu.

**Writing – review & editing:** Meng Li.
